# Zinc transporter SLC39A13/ZIP13 facilitates the metastasis of human ovarian cancer cells via activating Src/FAK signaling pathway

**DOI:** 10.1186/s13046-021-01999-3

**Published:** 2021-06-21

**Authors:** Xinxin Cheng, Jie Wang, Chunling Liu, Tianduo Jiang, Ningzhi Yang, Dan Liu, Huanhuan Zhao, Zhelong Xu

**Affiliations:** 1 Department of Physiology and Pathophysiology, Tianjin Medical University, 300070 Tianjin, China; 2 Department of Pathology, North China University of Science and Technology Affiliated Tangshan People’s Hospital, 063000 Tangshan, China

**Keywords:** Ovarian cancer, Zinc transporter, SLC39A13/ZIP13, Src/FAK, Metastasis, Zinc homeostasis

## Abstract

**Background:**

Zinc transporters have been found to be associated with the pathogenesis of numerous human diseases including cancer. As the most lethal gynecologic malignancy, ovarian cancer is characterized by rapid progression and widespread metastases. However, the function and underlying mechanism of zinc transporters in ovarian cancer metastasis remain unclear.

**Methods:**

The relationship between zinc transporter gene expressions and clinical outcomes of ovarian cancer was assessed with the online database Kaplan-Meier plotter (http://kmplot.com/analysis/). Immunohistochemistry was performed to investigate the prognostic importance of ZIP13. The expression of ZIP13 in ovarian cancer cell lines was depleted to explore its effect on proliferation, adhesion, migration, and invasion both in vitro and in vivo assays. RNA-Seq, quantitative RT-PCR, and western blot analysis were performed to explore ZIP13-regulated downstream target genes.

**Results:**

The expressions of several zinc transporters were highly associated the clinical outcomes of ovarian cancer patients. Among them, high ZIP13 expression was an independent prognostic factor for poor survival in patients with ovarian cancer. ZIP13 knockout suppressed the malignant phenotypes of ovarian cancer cells both in vitro and in vivo. Further investigation revealed that ZIP13 regulated intracellular zinc distribution and then affected the expressions of genes involved in extracellular matrix organization and cytokine-mediated signaling pathway. This led to the activation of Src/FAK pathway with increased expressions of pro-metastatic genes but decreased expressions of tumor suppressor genes.

**Conclusions:**

ZIP13 is shown to be a novel driver of metastatic progression by modulating the Src/FAK signaling pathway, which may serve as a promising biomarker for prognostic evaluation and targeted therapy in ovarian cancer.

**Supplementary Information:**

The online version contains supplementary material available at 10.1186/s13046-021-01999-3.

## Background

Ovarian cancer is the most lethal gynecological malignancy in women. Due to the lack of specific symptoms and effective methods for early detection, approximately 75 % of ovarian cancer patients are diagnosed at an advanced stage (III/IV). Despite advances in surgery and chemotherapy, the 5-year survival rate of women with widespread metastases is less than 30 % [1, 2]. Tumor metastasis is the major clinical challenge in the management of ovarian cancer. Peritoneal metastasis is frequently observed in ovarian cancer patients and is responsible for the poor prognosis of these patients [3]. Therefore, it is critical to elucidate the underlying molecular mechanisms and identify novel metastasis-related molecules for ovarian cancer, which may lead to potential therapeutic targets.

Zinc is a critical structural component of plenty of proteins, including enzymes, transcription factors, and signaling molecules. Zinc deficiency can lead to stunted cell growth and serious metabolic disorders, whereas excess zinc can also be cytotoxic [4]. As a result, mammalian cells have evolved a complex zinc transport network to maintain intracellular zinc homeostasis [5]. Two families of transporters, ZIPs (Zrt/Irt-like protein, SLC39A) and ZnTs (SLC30A), are required for the regulation of zinc uptake, efflux, and intracellular compartmentalization.

Accumulative studies support the fact that the disturbance of zinc homeostasis is associated with various diseases, including Alzheimer’s disease, diabetes, and cancer. Intracellular zinc dyshomeostasis induced by the dysfunction of zinc transporters, especially ZIP transporters, is found to be involved in the development and progression of several human malignancies [6, 7]. However, there is still limited mechanistic information available about the relationships between zinc dyshomeostasis and ovarian cancer. Some previous reports demonstrate that zinc induces apoptosis in ovarian cancer cells [8, 9]. A recent study also shows that zinc contributes to ovarian tumor metastasis by promoting epithelial-mesenchymal transition (EMT) through a MTF-1 dependent pathway [10]. Although ZIP4 is found to be a new and important cancer stem cell (CSC) regulator in ovarian cancer [11, 12], the prognostic significances and functions of other zinc transporters in ovarian cancer have not yet been fully explored.

ZIP13, a member of the SLC39A/ZIP family, is mainly localized in the Golgi apparatus. It has been shown to play important roles in the development of bone, tooth and connective tissues [13]. The pathogenic mutations of ZIP13 caused rapid degradation of functional ZIP13 via the valosin-containing protein (VCP)-linked ubiquitin proteasome pathway. This progress may interfere with collagen maturation and ultimately contribute to the development of a very rare autosomal recessive disease named Spondylocheirodysplastic-Ehlers-Danlos syndrome (SCD-EDS, OMIM 612350) [14]. Moreover, ZIP13 is a recently-found dermis zinc transporter, and its dysfunction often leads to severe dermal disorders [15]. The expression of ZIP13 is epigenetically suppressed in fibrosarcoma, leading to autophagy impairment and ultimately hypersensitivity to nutrient deficiency [16]. However, there is little knowledge about the potential roles of ZIP13 in cancers, especially ovarian cancer.

In the present study, we comprehensively assessed the associations between ZIP family and clinical outcomes in ovarian cancer, and identified ZIP13 as an independent prognostic factor in patients with ovarian cancer. Further investigation revealed that ZIP13 promoted cell proliferation, invasion, adhesion and metastasis both in vitro and in vivo. The underlying mechanisms were involved in the disruption of intracellular zinc distribution and the activation of the Src/FAK pathway, which ultimately led to ovarian cancer metastasis. This may provide a novel prognostic biomarker and therapeutic target for ovarian cancer patients.

## Materials and methods

### Cell lines and cell culture

Human ovarian cancer cell lines (SKOV-3 and HO-8910 PM) and HEK 293T cells were purchased from the Cell Resource Center, Peking Union Medical College (which is the headquarters of National Infrastructure of Cell Line Resource, NSTI). The ovarian cancer cell lines were cultured in RPMI 1640. HEK 293T cells were cultured in DMEM. All cells were cultured in medium supplemented with 10 % fetal bovine serum (FBS) (Invitrogen, Carlsbad, CA) at 37 °C in a humidified incubator containing 5 % CO_2_.

### Data mining and analyses

An online database Kaplan-Meier plotter (http://kmplot.com/analysis/) was used to investigate the association between zinc transporters mRNA levels and survival of ovarian cancer patients (data updated July 22, 2020). The Kaplan-Meier plotter is capable to evaluate the effect of 54k genes on patient clinical outcomes in numerous cancer types including breast, ovarian, lung, and gastric cancer [17]. To analyze the prognostic value of ZIP transporters in ovarian cancer, only datasets with available raw microarray gene expression data, clinical survival information, and at least 20 patients were included. The gene expression data and survival information of these ovarian cancer patients were publicly available at the Gene Expression Omnibus (GEO) (http://www.ncbi.nlm.nih.gov/geo/) and the Cancer Genome Atlas (TCGA) (http://cancergenome.nih.gov/). The patients in datasets were classified into two groups according to auto select best cutoff (high vs. low expression). The detailed clinical characteristics of ovarian cancer patients used in the analysis were listed in Table S1. Overall survival (OS, time from randomization until death from any cause) and progression-free survival (PFS, time from randomization to progression or death from any cause) were employed as the major indicators for a patient’s prognosis. The prognostic values of SLC39A/ZIP genes associated with clinicopathological features and risk factors were also explored in this database. The hazard ratio (HR) with 95 % confidence intervals (CI) and log rank *P* value were calculated and shown on the web pages. *P* value of < 0.05 was considered to be statistically significant. The cBioPortal database (http://www.cbioportal.org) was used to conduct gene expression correlation analysis between ZIP13 and target genes in ovarian cancer samples from TCGA.

### Tissue microarray assay and immunohistochemical staining

Tissue microarray (TMA) chips of ovarian cancer were purchased from Shanghai Outdo Biotech Company (Shanghai, China) (No.HOvaC154Su01) and Shanxi Alenabio Biotechnology (Xi’an, China) (No.DC-Ova11039). All specimens on the HOvaC154Su01 chip were well documented with complete follow-ups for periods from 5 to 9 years. Tumor staging was evaluated according to the TNM classification of malignant tumors. For immunohistochemistry (IHC) analysis, the TMAs were incubated with anti-ZIP13 (ab106586, Abcam) overnight at 4 °C after deparaffinization, rehydration, antigen retrieval and blocking the endogenous peroxidase activity. The primary antibody was replaced with PBS in sections used as negative controls. Following washing, slides were incubated in a HRP-conjugated secondary antibody at room temperature for 1 h. The slides were visualized for DAB staining, and counterstained with hematoxylin.

### Evaluation of immunohistochemical results

Stained TMA slides were evaluated by two independent pathologists who were blinded for clinicopathological parameters. The IHC score was used to evaluate ZIP13 expression in ovarian cancer tissues. It was calculated by multiplying the staining intensity and the percentage of positive staining. For statistical analysis, IHC score > 4 was regarded as high expression.

### Lentivirus infection

Lentivirus CRISPR constructs targeting ZIP13 were made using the LentiCRISPRv2 vector (Addgene Plasmid 52961) following the published protocol [18]. Briefly, the LentiCRISPRv2 vector was digested with BsmBI (R0580S, NEB, USA) and ligated with annealed sgRNA target sequences. Each construct was sequenced to verify correct incorporation into the lentiCRISPRv2 vector. To generate lentivirus for LentiCRISPRv2 constructs, HEK 293T cells at 80 % confluency were transfected with recombinant vectors and packaging vectors pMD2G and psPAX2 using Lipofectamine 3000 (Invitrogen, CA, USA). Viral supernatants were harvested 48 h later, centrifuged at 500 g for 5 min to remove debris, and filtered through a 0.45 μm cellulose filter (Millipore, MA, USA). The viral supernatants from different target sgRNAs were mixed and applied to ovarian cancer cells with 8 µg/ml polybrene. Stably infected cells were selected using 2 µg/ml puromycin (Sigma, MO, USA) for 7 days. Knockout efficiency was determined by western blot analysis and DNA sequencing. The sgRNA target sequences were listed in Table S2.

### Cell proliferation and colony formation assay

Cell proliferation assay was performed using cell counting kit 8 (CCK-8, Dojindo, Japan) according to the manufacturer’s instructions. At 0, 24, 48, 72 and 96 h, CCK-8 solution was added to each well and incubated for 1 h. Optical density values at wavelength 450 nm were measured by a microplate reader. All conditions were tested in six replicates. To determine cell colony formation efficiency, 1000 cells were plated in 6-well plates and cultured for 2 weeks. Then the colonies were washed by PBS, followed by fixing in 4 % polyformaldehyde, and staining in 0.5 % crystal violet. Each experiment was independently conducted at least three times. The colony number was counted under an optical microscope.

### Wound healing assay

The wound healing assay was conducted according to protocols described elsewhere [19]. Cells were seeded in 6-well plates at the density of 2 × 10^5^ cells/ml. A vertical wound was made through confluent monolayer cells by using a sterile pipette tip, followed by washing with medium to remove cell debris. Subsequently, the cells were cultured in the serum-free medium for 48 h. Wound areas were photographed with a phase-contrast microscope (Olympus, Tokyo, Japan) and quantified using Image J software.

### Transwell assay

The transwell invasion and migration assays were conducted using the 24-well transwell chambers (8 μm, Corning, USA). For migration assay, cells were collected and resuspended in serum-free medium. Then the upper chambers were plated with cell suspensions at the density of 5 × 10^4^ cells/ml, while 600 µL RPMI medium supplemented with 20 % FBS was placed into the lower chambers as the chemoattractant. After 24 h of incubation at 37 °C, cells remaining on the upper membrane were removed carefully, and the migrated cells adherent to the bottom surface were fixed with 4 % paraformaldehyde and stained with 0.5 % crystal violet. For the invasion assay, the transwell chambers were pre-coated with Matrigel (Corning, NY, USA), and other procedure was the same as described above. The experiments were repeated at least three times. The migratory or invasive cells were visualized and counted in at least three random fields under a microscope (Olympus, Tokyo, Japan).

### Cell adhesion assay

Adhesion assays were performed by resuspending ovarian cancer cells in serum-free medium and stained with 5 µM Calcein-AM (Invitrogen, Carlsbad, CA) for 30 min at 37 °C. After incubation, cells were washed and collected. Calcein-AM-labeled cells were seeded into 96-well plates pre-coated with either 10 µg/ml Matrigel (Corning, NY, USA) or 50 µg/ml fibronectin (Sigma, USA) at a density of 1 × 10^5^ cells per well and allowed to adhere at 37 °C for 30 min. Then the cells were washed twice with PBS to remove non-adherent cells. Fluorescent signal of the adherent Calcein-AM-labeled cells was imaged under the microscope (Olympus, Tokyo, Japan) and quantified with a fluorescent plate reader (Molecular Devices, Sunnyvale, CA). All experiments were performed at least three times.

### RNA isolation, reverse transcription PCR, and quantitative real-time PCR (RT-PCR)

Total RNA was extracted with TRIzol reagent (Invitrogen, CA, USA) according to the manufacturer’s instructions. Complementary DNAs (cDNA) were synthesized using GoScript™ Reverse Transcription System (Promega, WI, USA). Quantitative RT-PCR was performed using the SYBR Green qPCR Master Mix (Bio-Rad, CA, USA) with a CFX96 Touch™ Real-Time PCR Detection System (Bio-Rad, CA, USA). GAPDH was chosen as a reference gene. The mRNA expression levels of the target genes were quantified by using the 2^−ΔΔCt^ method. The primer sequences were shown in Table S3.

### Western blot analysis

Cultured cells were lysed in RIPA buffer (Thermo Scientific Pierce, MA, USA) in the presence of a protease inhibitor cocktail (Roche Applied Science, IN, USA). Protein concentration was quantified using a BCA protein assay kit (Thermo Scientific Pierce, MA, USA). Then proteins were separated in SDS-PAGE, followed by transferring onto an Immobilon-P Transfer membrane (Merck Millipore, MA, USA). Immunoblots were performed by standard protocol. Chemiluminescence were detected by the enhanced chemiluminescence (ECL) kit (Millipore, MA, USA).

The primary antibodies used in this study were: GAPDH (5174, Cell Signaling Technology), β-Tubulin (YM3030, ImmunoWay), Vimentin (5741, Cell Signaling Technology), N-cad (4061, Cell Signaling Technology), Snail (3879, Cell Signaling Technology), Slug (9585, Cell Signaling Technology), E-cad (3195, Cell Signaling Technology), c-Jun (24909-1-AP, Proteintech), ERK (4695, Cell Signaling Technology), pERK (4377, Cell Signaling Technology), STAT3 (4904, Cell Signaling Technology), pSTAT3 Ser727 (9134, Cell Signaling Technology), pSTAT3 Tyr705 (9145, Cell Signaling Technology), Src (2108, Cell Signaling Technology), pSrc (2105, Cell Signaling Technology), FAK (13009, Cell Signaling Technology), pFAK Tyr397 (8556, Cell Signaling Technology), pFAK Tyr925 (3284, Cell Signaling Technology), Anti-Mouse IgG (7076, Cell Signaling Technology), Anti-rabbit IgG (7074, Cell Signaling Technology).

### Experimental peritoneal metastatic model

All mice were housed a specific pathogen-free (SPF) facility with free access to autoclaved food and water. All animal protocols were approved by the Animal Care and Use Committee of the Tianjin Medical University. Human ovarian cancer cells were collected and inoculated into the peritoneal cavities of 5-week-old BALB/c nude mice (1 × 10^6^ cells per mouse, *n* = 6 per group). The mice were sacrificed 2 months after cell inoculation. All visible peritoneal tumors were dissected, counted and weighed. The ascites production was also measured for the volume.

### RNA sequencing (RNA-seq) and data analysis

The RNA-seq was used to analyze the gene expression profile after ZIP13 knockout. The Library construction and sequencing were performed at Beijing Genomics Institute (BGI) on the BGISEQ-500 platform (Shenzhen, China). Gene expression was quantified using RSEM tool. The NOISeq method was used to screen for differentially expressed genes (DEGs) between the two groups. The significance of DEGs were determined by the threshold with a fold change > 2 and *P* < 0.05. The GO and KEGG pathway enrichment analysis were conducted as previously described [20].

### Statistical analysis

The SPSS 21.0 (Chicago, IL) and Graphpad Prism (San Diego) were used for statistical analysis. Data were shown as mean ± SEM. The association between ZIP13 expression and the clinicopathological variables was assessed by χ^2^ test. Survival curves were plotted using the Kaplan-Meier method to assess the effects of ZIP13 expression on survival with significance evaluated by log-rank test. The Cox proportional hazards regression model was used for both univariate and multivariate analysis of survival and for estimating HR with 95 % CI. Comparisons between two groups were evaluated by the Student’s t-test. For comparisons involving more than two groups, one-way analysis of variance was performed, followed by pair-wise t tests with Bonferroni correction. Statistical significance was set at *P* < 0.05.

## Results

### The expressions and clinical outcomes of ZIP transporters in ovarian cancer

**Fig. 1:**
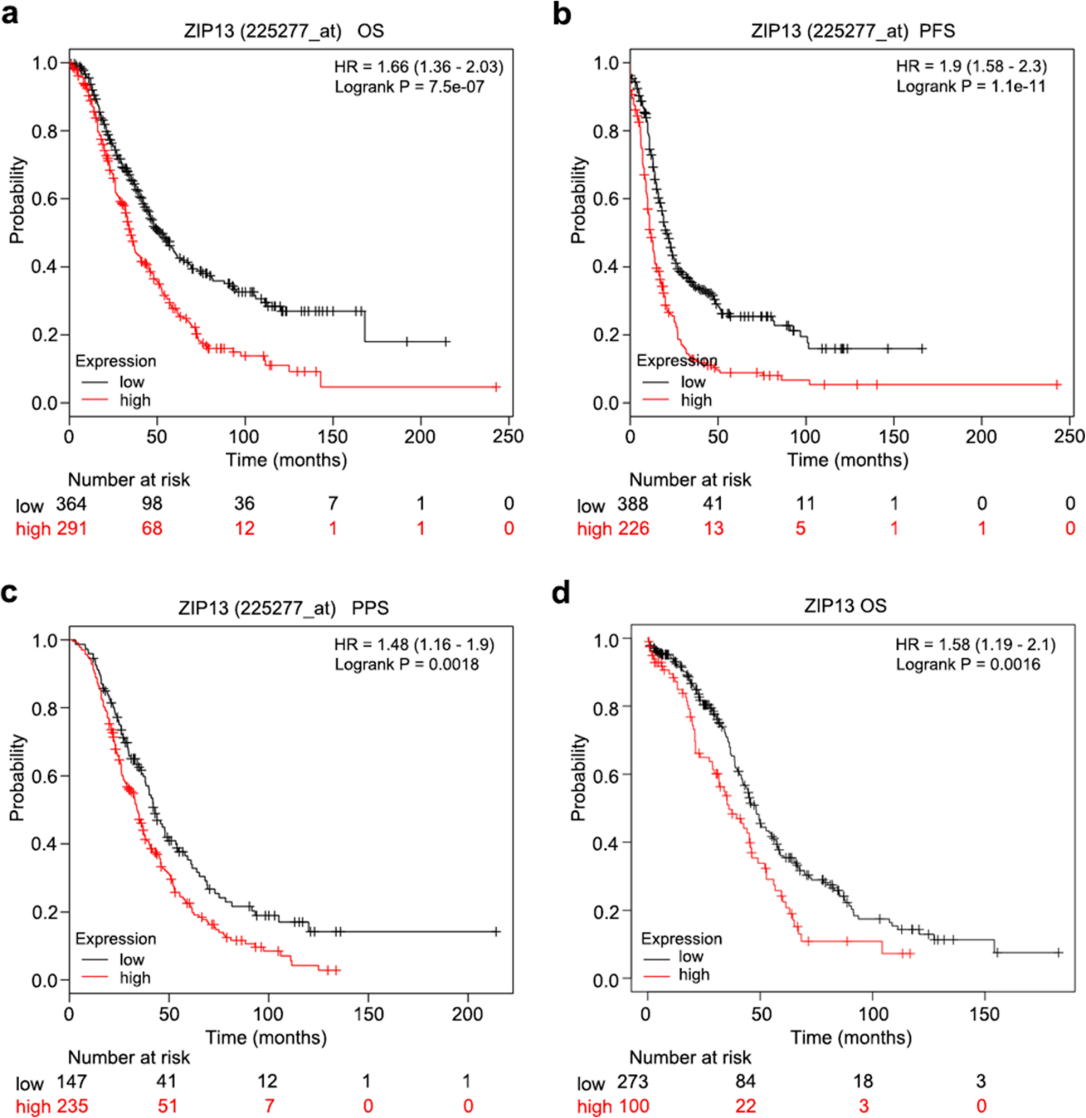
Kaplan-Meier survival analysis of ZIP13 gene expression in ovarian cancer patients. **a-c**. Overall survival (OS) (**a**), progression-free survival (PFS) (**b**) and post-progression survival (PPS) (**c**) curves of ZIP13 in patients with ovarian cancer by using Kaplan-Meier plotter. **d**. Kaplan-Meier survival analysis of ZIP13 expression in The Cancer Genome Atlas (TCGA) cohort

The mRNA expressions of ZIP transporters were analyzed by the online database Kaplan-Meier plotter (http://kmplot.com/analysis/) to evaluate their prognostic values in ovarian cancer tissues. As shown in Table 1, Fig. 1a and b, high expressions of ZIP5, ZIP10, ZIP12, ZIP13 and ZIP14 were significantly associated with unfavorable overall survival (OS) and progression-free survival (PFS) in ovarian cancer patients. Given that the serous ovarian cancer accounts for 70 % of all ovarian tumors, we also evaluated the prognostic values of ZIPs in patients with serous ovarian cancer (Table S4). Although the expressions of most ZIP transporters were associated with either OS or PFS, only elevated mRNA levels of ZIP3, ZIP5, ZIP12 and ZIP13 were closely related to poor OS and PFS in serous ovarian cancer patients. In addition, another dataset from the Cancer Genome Atlas (TCGA, RNA-seq data) was employed to validate the prognostic impact of these potential genes (Fig. 1d and Fig. S1). The results consistently supported that high expression levels of ZIP12 and ZIP13 expression predicted poor OS in ovarian cancer patients.

**Table 1:** The association of ZIP transporters with overall survival (OS) and progression-free survival (PFS) in ovarian cancer patients

Genes	OS			PFS		
	**Patients Number**	**HR (95 % CI)**	**Logrank** ***P***	**Patients Number**	**HR (95 % CI)**	**Logrank** ***P***
ZIP1	1656	1.13 (0.97–1.33)	0.12	1435	1.16 (1.01–1.33)	0.037
ZIP2	1656	0.93 (0.80–1.07)	0.31	1435	0.89 (0.78–1.02)	0.089
ZIP3	655	1.18 (0.96–1.45)	0.11	614	0.86 (0.70–1.05)	0.14
ZIP4	1656	0.92 (0.80–1.05)	0.22	1435	0.92 (0.81–1.05)	0.2
ZIP5	655	1.32 (1.08–1.62)	0.0066	614	1.48 (1.23–1.79)	3.50E-05
ZIP6	1656	1.08 (0.94–1.25)	0.29	1435	0.84 (0.72–0.97)	0.019
ZIP7	1656	0.78 (0.67–0.90)	0.00058	1435	1.17 (1.03–1.33)	0.014
ZIP8	1656	1.06 (0.93–1.21)	0.37	1435	0.87 (0.75–1.01)	0.071
ZIP9	655	1.18 (0.94–1.48)	0.15	614	0.89 (0.74–1.08)	0.23
ZIP10	655	1.30 (1.06–1.60)	0.012	614	1.28 (1.06–1.55)	0.009
ZIP11	655	1.11 (0.90–1.38)	0.32	614	0.85 (0.70–1.04)	0.11
ZIP12	655	1.24 (1.01–1.54)	0.044	614	1.50 (1.22–1.84)	9.10E-05
ZIP13	655	1.66 (1.36–2.03)	7.50E-07	614	1.90 (1.58–2.30)	1.10E-11
ZIP14	1656	1.25 (1.10–1.42)	0.00066	1435	1.27 (1.12–1.44)	2.00E-04

### ZIP13 correlates with tumor progression and poor prognosis in ovarian cancer

In light of the most significant association of ZIP13 with the survival of ovarian cancer patients (*P* = 7.50E-07 for OS, and *P* = 1.10E-11 for PFS), we thus assumed that ZIP13 was one of the most promising ZIP transporters in ovarian cancer. In addition to OS and PFS, further analysis revealed that high mRNA expression of ZIP13 was also remarkably associated with reduced post-progression survival (PPS) (Fig. 1c). Then we explored the prognostic value of ZIP13 in ovarian cancer with different clinical stages and pathological grades. As shown in Table S5, ovarian cancer patients with elevated ZIP13 mRNA expression had worse OS in either early-stage cases or late-stage cases. Additionally, the upregulation of ZIP13 mRNA was related with worse PFS and PPS only in patients with late-stage. Similar results were also observed in ovarian cancer patients with different pathological grades (Table S6).

**Fig. 2:**
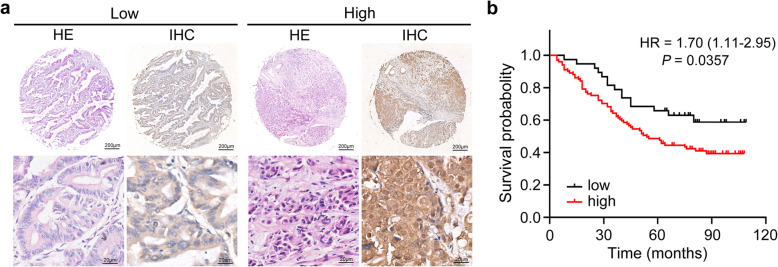
High ZIP13 protein level in ovarian cancer is associated with poor outcome. **a** IHC analysis of ZIP13 expression. **b** Kaplan-Meier survival analysis of OS in ovarian cancer samples from a tumor tissue microarray (TMA)

To further substantiate the prognostic importance of ZIP13 in ovarian cancer progression, we assessed ZIP13 protein expression on TMA chips. Representative images of ZIP13 staining were shown in Fig. 2a. As shown in Table 2, high level of ZIP13 protein expression was significantly associated with serous carcinoma (*P* = 0.031), lymphatic metastasis (*P* = 0.004), and distant metastasis (*P* = 0.002). Consistent with the observations obtained from online database, Kaplan-Meier analysis revealed that patients with high expression of ZIP13 protein had a significantly shorter OS survival than those with low ZIP13 expression (*P* = 0.0357, Fig. 2b). Furthermore, the univariate Cox regression analysis suggested that patient age ≥ 60 years, serous histological subtype, FIGO stage (*P* < 0.001), tumor size (*P* < 0.001), and ZIP13 protein expression (*P* = 0.04) were poor prognostic factors for OS in ovarian cancer patients (Table 3). Further multivariate analysis revealed that FIGO stage (*P* < 0.001), tumor size (*P* < 0.001), and ZIP13 protein expression (*P* = 0.046) were independent prognostic factors for OS in ovarian cancer patients. In addition, ZIP13 expression was also assessed by IHC analysis in another cohort with normal ovarian tissues. As shown in Fig. S2, the result revealed that ZIP13 is aberrantly overexpressed in ovarian cancer tissues. Collectively, these results suggested that ZIP13 could serve as a promising biomarker for ovarian cancer diagnosis and prognosis.

**Table 2:** Association of ZIP13 expression with clinicopathological characteristics in ovarian cancer

Clinicopathological characteristics	Total	ZIP13 expression		χ^**2**^	***P***
	***N*** = 139	Low	High		
Age, years				0.64	0.424
<60	103	30	73		
≥60	36	8	28		
Sample type				1.802	0.179
Primary tumor	126	37	89		
Recurrence/metastasis tumor	13	1	12		
Histological/Histologic subtype				4.662	0.031
Serous carcinoma	86	18	68		
Non-serous carcinoma	53	20	33		
Tumor size, cm				1.522	0.217
<5	40	8	32		
≥5	99	30	69		
Primary tumor stage				1.999	0.157
T1/T2	39	14	25		
T3/T4	100	24	76		
Lymphatic metastasis				8.499	0.004
No	99	34	65		
Yes	40	4	36		
Distant metastasis				9.306	0.002
No	107	36	71		
Yes	32	2	30		
FIGO stage				1.999	0.157
I/II	39	14	25		
III/IV	100	24	76

**Table 3:** Univariate and multivariate COX regression analyses of OS in ovarian cancer patients

Clinicopathological characteristics	Univariate		Multivariate	
	HR (95% CI)	*P*	HR (95%)	*P*
Age, years	1.79 (1.11–2.88)	0.016	0.97 (0.60–1.58)	0.9
≥ 60 vs < 60				
Histological subtype	1.82 (1.10–3.02)	0.019	0.99 (0.57–1.72)	0.984
Serous vs Non-serous				
Tumor size (cm)	4.84 (2.38–9.84)	< 0.001	5.58 (2.67–11.64)	< 0.001
≥ 5 vs < 5				
FIGO stage	10.56 (3.85–28.97)	< 0.001	10.44 (3.75–29.04)	< 0.001
III/IV vs I/II				
ZIP13	1.81 (1.02–3.19)	0.04	1.86 (1.10–3.41)	0.046
High vs Low				

### ZIP13 suppression inhibits ovarian cancer metastasis in vivo

To investigate the functions of ZIP13 in ovarian cancer biology, we utilized the CRISPR/Cas9 genome editing technology to deplete ZIP13 expression in ovarian cancer cell lines and investigated the potential roles of ZIP13 in regulating metastasis-relevant traits. The knockout efficiency was confirmed by DNA sequencing analysis and western blots (Fig. S3).

**Fig. 3:**
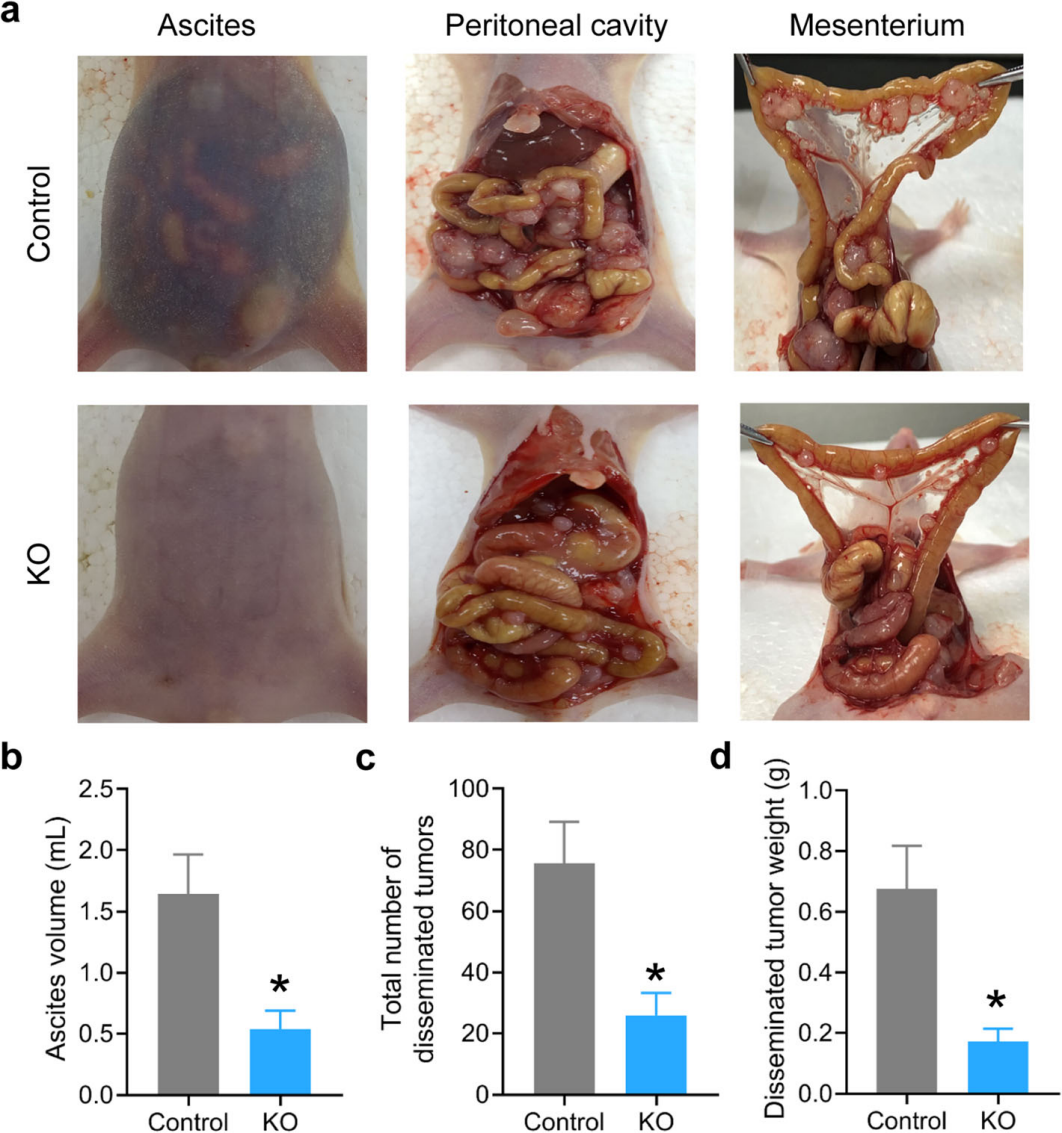
ZIP13 knockout suppresses peritoneal dissemination of ovarian cancer cells in vivo. **a** SKOV-3 cell suspensions were injected into peritoneal cavities of BALB/c nude mice (*n* = 6 mice per group). Representative photos of ascites, tumor formation at the peritoneal cavity and mesenterium were shown. **b** The ascites was collected and measured. **c**, **d.** The disseminated tumors in the abdominal cavities were estimated and quantified. *P < 0.05

We first evaluated the effect of ZIP13 knockout on peritoneal dissemination potential of ovarian cancer cells in vivo. A peritoneal metastasis model was generated by intraperitoneal injection ovarian cancer cell suspensions into cohorts of BALB/c nude mice. The visible peritoneal tumors and ascites were collected and measured (Fig. 3a). Consistent with the findings in vitro, mice injected with ZIP13-depleted ovarian cancer cells exhibited lower ascites formation at 2 months post-inoculation (Fig. 3b). The tumor burdens in the peritoneum and mesentery were also significantly lower in the ZIP13-depleted group than that of the control group, as indicated by the number of tumor nodules and total tumor weight (Fig. 3c and d). In support of these findings, ZIP13 knockout in HO-8910 PM cells also significantly inhibited total ovarian peritoneal tumor mass and tumor number in mice injected with these cells (Fig. S4). Collectively, these in vivo observations strongly suggested that ZIP13 is essential for ovarian tumor metastasis.

### ZIP13 maintains malignant phenotypes of ovarian cancer cells

**Fig. 4:**
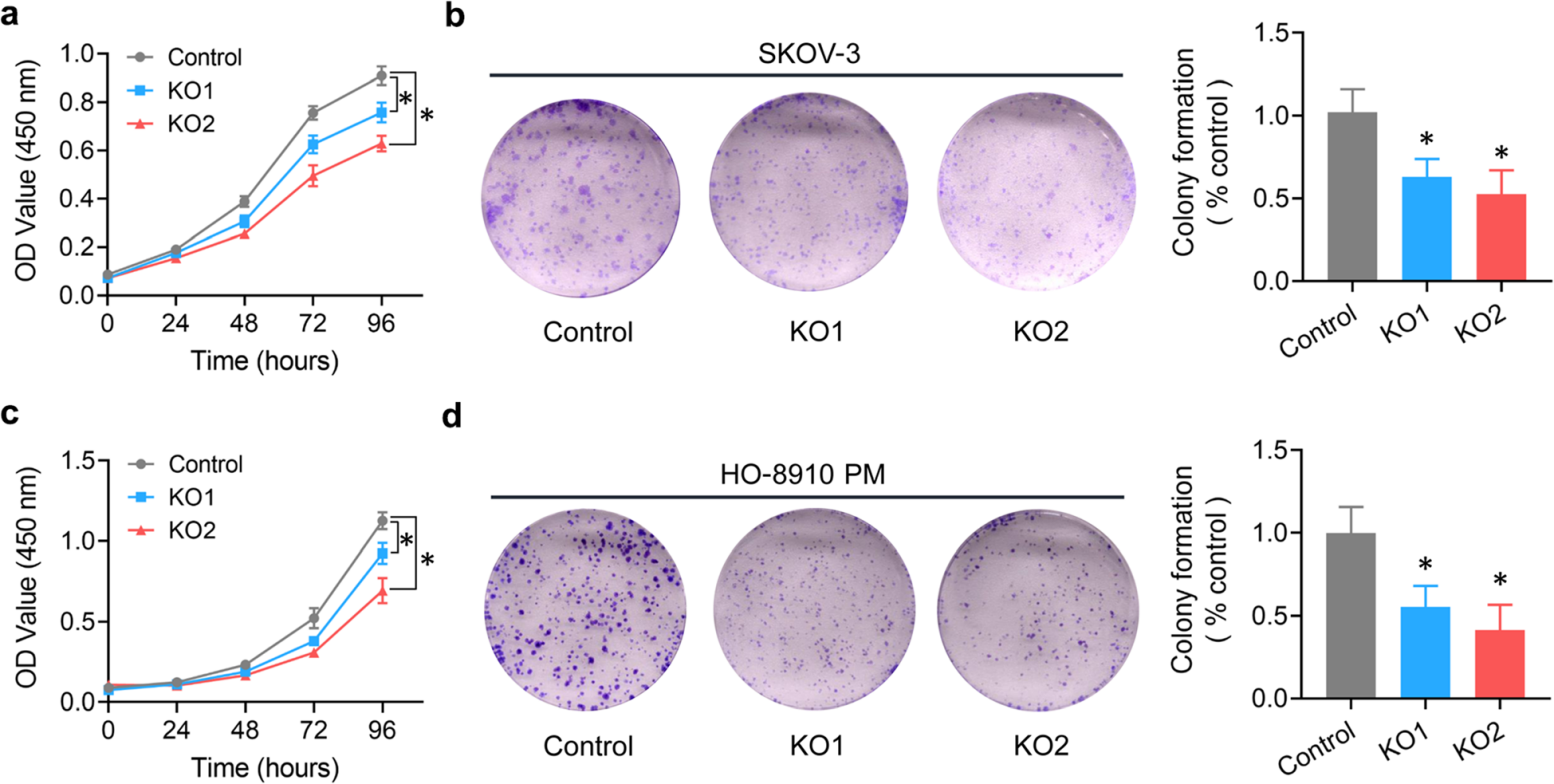
ZIP13 knockout inhibits the growth of ovarian cancer cells in vitro. **a, c.** Cell proliferation assay was performed using CCK-8. At 0, 24, 48, 72 and 96 h, CCK-8 solution was added to each well and OD450 values were measured by a microplate reader. All conditions were tested in six replicates. **b, d.** SKOV-3 and HO-8910 PM cells were plated in 6-well plates and cultured for 2 weeks. Then the colonies were fixed and stained. The colony number was counted under an optical microscope. Each experiment was independently conducted at least three times. *P < 0.05

We next evaluated the in vitro effect of ZIP13 knockout on the malignant phenotypes of human ovarian cancer cells. CCK-8 assay and colony formation assay were used to examine the effect of ZIP13 on cell proliferation. As shown in Fig. 4a, knockout of ZIP13 in SKOV-3 cells significantly dampened the cell growth ability comparing to the control. Moreover, ZIP13 knockout also inhibited colony-forming ability (Fig. 4b). Similar results were also observed with HO-8910 PM cells (Fig. 4c and d). Together, these results suggested that ZIP13 knockout inhibited cell growth in ovarian cancer cells.

**Fig. 5:**
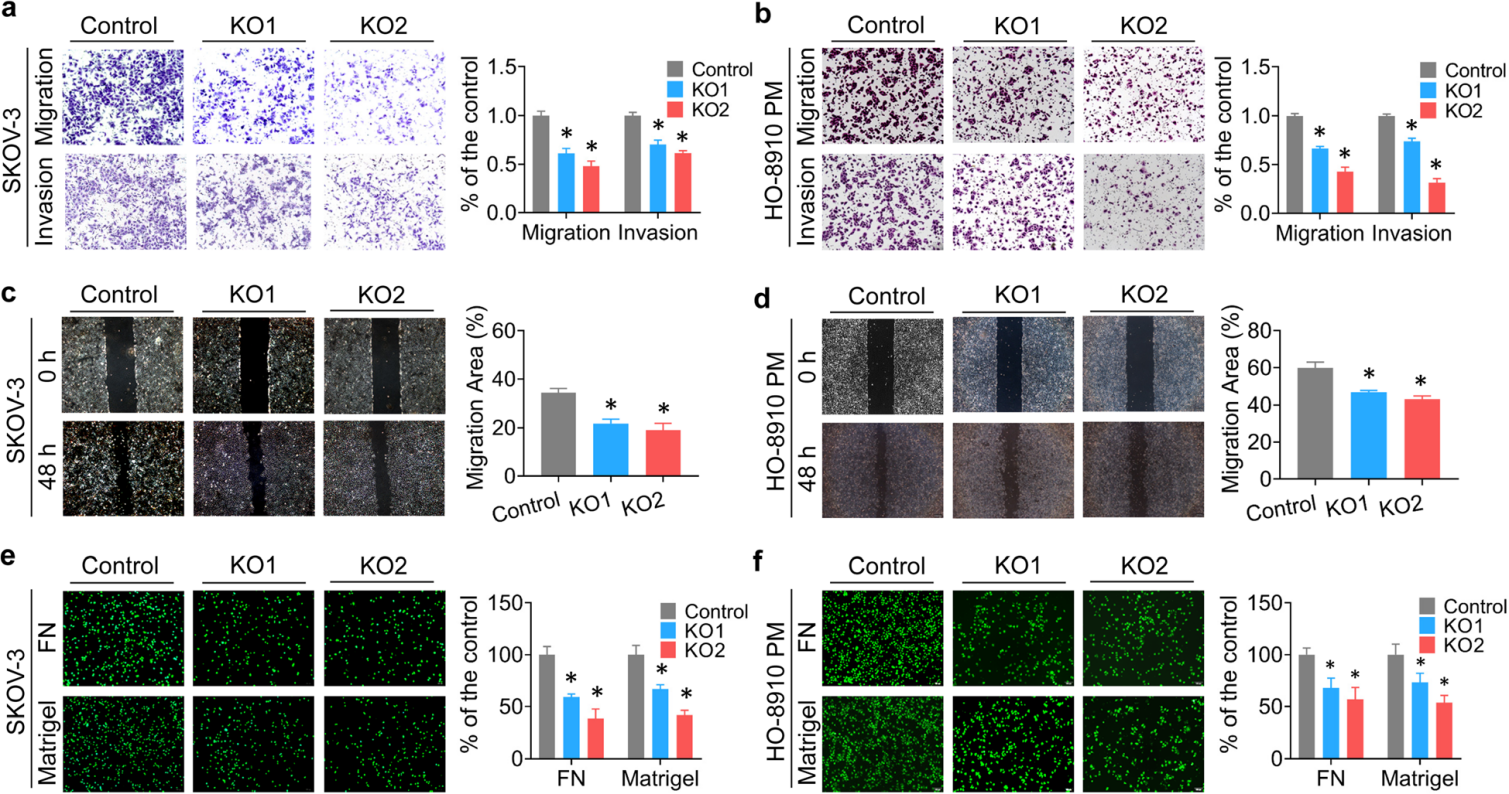
ZIP13 promotes ovarian cancer cell migration, invasion and adhesion in vitro. **a, b.** The transwell invasion and migration assays were conducted using the 24-well transwell chambers. The migratory or invasive cells were visualized and counted in at least three random fields under a microscope. **c, d.** A vertical wound was made through confluent monolayer cells. Then the wound areas were photographed and quantified after 48 h. **e, f.** Calcein-AM-labeled cells were seeded into 96-well plates pre-coated with either Matrigel or fibronectin (FN), and allowed to adhere at 37 °C for 30 min. The fluorescent signal of the adherent cells was imaged under the microscope and quantified with a fluorescent plate reader. Results indicated were mean values of three independently repeated experiment. *P < 0.05

Cell invasion, motility, and adhesion are the key features of the metastasis. Then we conducted a series of in vitro assays to explore the effects of ZIP13 on these malignant phenotypes. As shown in Fig. 5a and b, transwell assays demonstrated that ZIP13 knockout repressed the migratory and invasive abilities in SKOV-3 and HO-8910 PM cells. Similarly, knockout of ZIP13 markedly reduced ovarian cancer cell motility, as measured by wound healing assays (Fig. 5c and d). Furthermore, adhesion assays using plates coated with different extracellular matrix components, such as Matrigel and fibronectin (FN), were performed to evaluate the effect of ZIP13 on the adhesive behavior of ovarian cancer cells. We observed that adhesive ability was significantly decreased when ZIP13 expression was suppressed (Fig. 5e and f). Taken together, these vitro and vivo data indicated that ZIP13 was essential for maintaining the pro-metastasis traits of ovarian cancer cells.

### ZIP13 regulates cellular zinc distribution

It has been reported that ZIP13 is mainly located in Golgi apparatus, indicating that ZIP13 may function as an intracellular zinc transporter [13]. We stained cells with the zinc-specific fluorescent dye, Zinpyr-1 and found that cells with downregulation of ZIP13 showed more Zinpyr-1 stained vesicles and higher fluorescence intensity than the control cells (Fig. S5a). We also measured total cellular zinc levels by inductively coupled plasma mass spectrometry (ICP-MS) and observed that total cellular zinc levels were not significantly affected in ZIP13-depleted ovarian cancer cells (Fig. S5b). Therefore, we proposed that loss of ZIP13 may only alter the subcellular distribution of labile zinc.

### ZIP13 regulates metastasis-related genes in ovarian cancer

**Fig. 6:**
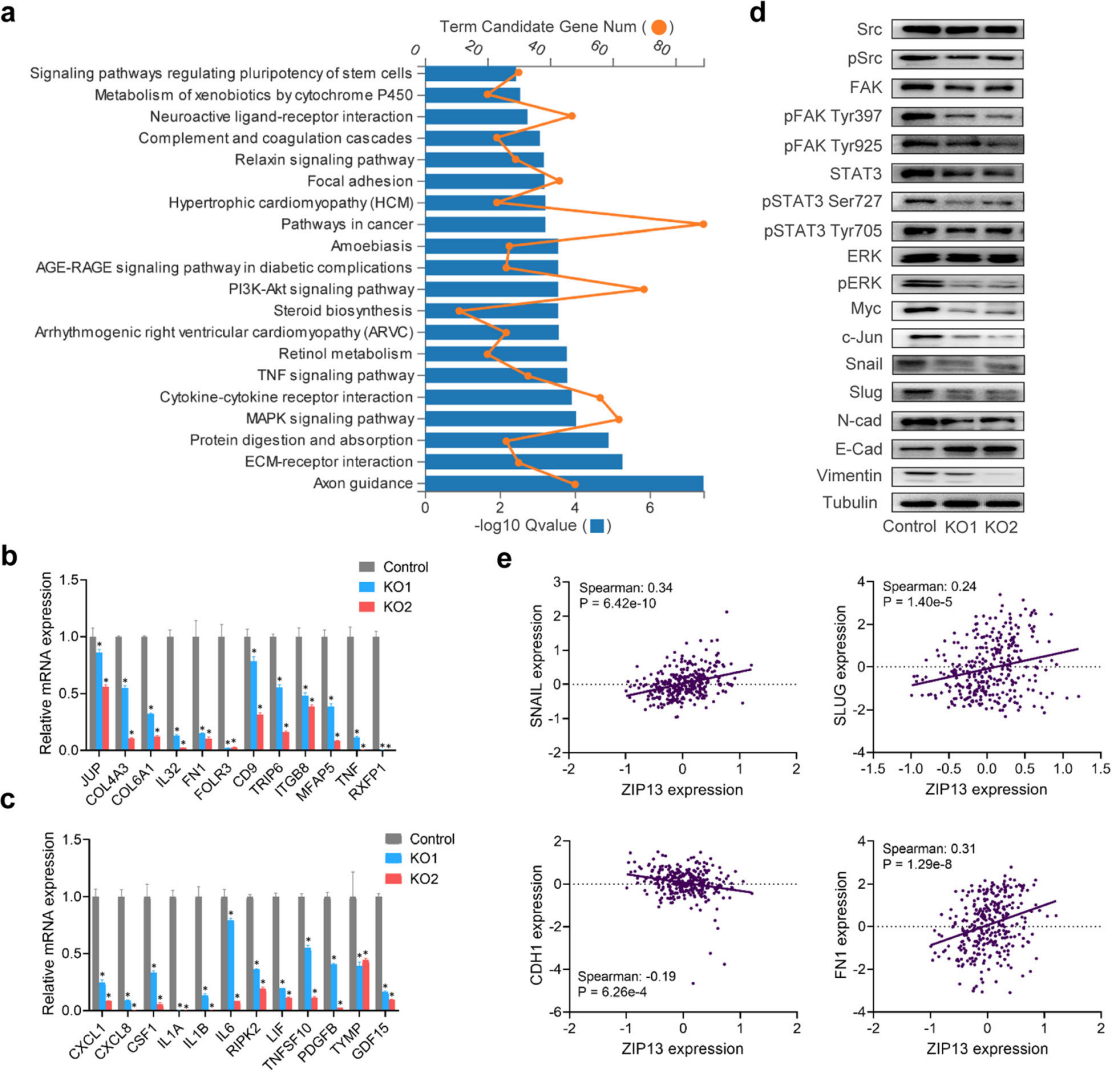
ZIP13 promotes ovarian cancer metastasis via the Src/FAK pathway.** a**. KEGG pathway enrichment analysis for ZIP13-regulated genes. **b, c**. Quantitative RT-PCR analysis of target genes identified in the RNA-seq. **d**. Western blot analysis of Src/FAK signaling pathway. **e**. Correlation analysis of the expression of ZIP13 and target genes in ovarian cancer tissues from TCGA. Spearman’s correlation coefficient and *P* values were shown for each analysis. *P < 0.05

To gain better insights into the underlying molecular mechanisms of ZIP13-mediated ovarian cancer metastasis, we performed RNA-seq analysis to explore differentially expressed genes after ZIP13 knockout. KEGG pathway analysis demonstrated that the target genes were enriched in various pathways, including extracellular matrixes (ECM)-receptor interaction, MAPK signaling pathway, cytokine-cytokine receptor interaction, TNF signaling pathway, and focal adhesion terms (Fig. 6a and Table S7). Similarly, GO analysis of biological processes demonstrated that DEGs were enriched in cell adhesion, extracellular matrix organization, regulation of signaling receptor activity and cytokine-mediated signaling pathway (Table S8). Molecular functions analysis revealed that gene terms related to protein binding, extracellular matrix structural constituent, cytokine activity and signaling receptor activity were also altered (Table S9). Subsequently, quantitative RT-PCR was used to confirm ZIP13-dependent expression of genes identified in RNA-seq analysis. The expressions of genes implicated in ECM and cell adhesion were blocked in ZIP13-depleted cells (Fig. 6b). In addition, a number of genes involved in regulation of signaling receptor activity and interaction were also affected when ZIP13 was knockout (Fig. 6c).

Focal adhesion kinase (FAK), also known as protein tyrosine kinase 2 (PTK2), is a non-receptor tyrosine kinase involved in cell proliferation, adhesion, migration and invasion. We further explored the role of ZIP13 in activation of Src/FAK signaling. As shown in Fig. 6d and Fig. S6, we detected a striking decrease of FAK expression in ZIP13-depleted cells. Additionally, phospho-Src (pSrc) and the Src/FAK downstream signals including STAT3, ERK, Myc, Snail, Slug, N-cad, and Vimentin, which are the markers of cell proliferation and migration, were all decreased. Consistent with this, we found that E-cad, an invasion-suppressor, was increased when ZIP13 was knockout (Fig. 6d). In addition, we further treated ovarian cancer cells with different concentrations of ZnCl_2_. As shown in Fig. S7, zinc activated Src/FAK pathway and regulated the expressions of some metastasis-related genes. These findings indicated that ZIP13 promoted the metastasis of ovarian cancer partly in a zinc-dependent manner.

Finally, we explored the gene expression profiles correlated with ZIP13 expression in clinical samples from TCGA. The results demonstrated that the genes that co-expressed with ZIP13 were enriched in focal adhesion, ECM-receptor interaction and PI3K-Akt signaling pathway (Table S10), which was in agreement with the previous results in our RNA-seq analysis. In addition, the expression of ZIP13 in the ovarian cancer samples was positively correlated with those pro-metastasis genes, such as Snail, Slug, FN1, and was negatively correlated with that tumor suppressor CDH1 (E-cad) gene (Fig. 6e and Fig. S8). These findings provided the evidence that ZIP13 facilitates ovarian cancer metastasis by regulating metastasis-related genes via Src/FAK signaling pathway.

**Fig. 7:**
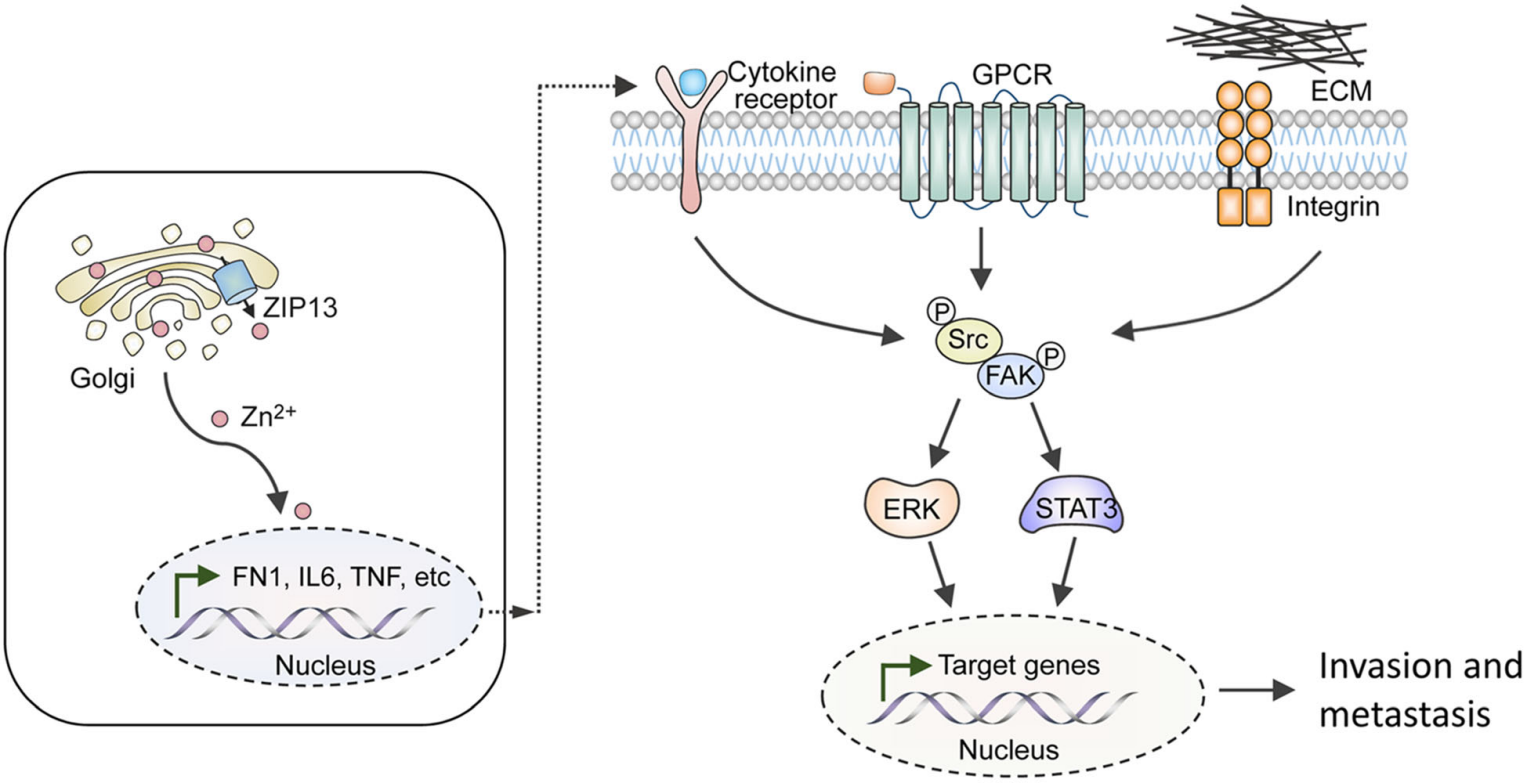
A schematic diagram illustrates the potential mechanisms by which ZIP13 promotes the metastasis of ovarian cancer. ZIP13 is located in the Golgi apparatus and regulates the intracellular distribution of zinc, which affects the expressions of genes involved in ECM organization and cytokine-mediated signaling pathway. This enable the activation of the Src/FAK pathway and downstream signaling cascade, thereby leading to the induction of targeted genes involved in cell invasion and tumour metastasis

Taken together, we identified ZIP13 as a novel driver of metastatic progression of ovarian cancer. The underlying mechanisms were involved in the disruption of intracellular zinc distribution and the activation of the Src/FAK pathway, which ultimately led to ovarian cancer metastasis (Fig. 7).

## Discussion

Metastasis is responsible for the high morbidity and mortality associated with ovarian cancer [3]. The elucidation of the molecular mechanisms underlying ovarian cancer metastasis will gain novel insights for the discovery of promising therapeutic targets for improving the treatment of ovarian cancer. Currently, zinc dyshomeostasis mediated by the ZIP transporters has been reported to be associated with the development and progression of human cancers, including some types of gynecological malignancies. For instance, Wei et al. found that ZIP7 played a critical role in cervical cancer carcinogenesis by regulating the expression of apoptotic and EMT biomarkers [21]. Recent studies conducted by Fan et al. also showed that ZIP4 was overexpressed in epithelial ovarian cancer, and participated in CSC-related cellular activities in high-grade serous ovarian cancer (HGSOC) [11]. In addition, ZIP4 was also an upstream regulator of several known CSC markers, including ALDH1, OCT4, SOX9, especially NOTCH3 [12]. Generally speaking, the clinical significance and potential roles of the ZIP family members have rarely been explored in gynecological cancers.

In the present study, we examined the expression profile of ZIP family members in ovarian cancer and investigated their associations with clinical outcomes. We observed that the upregulation of mRNA expressions of ZIP5, ZIP10, ZIP12, ZIP13 and ZIP14 was strikingly associated with unfavorable OS and PFS in patients with ovarian cancer. For patients with serous ovarian cancer, the elevated mRNA levels of ZIP3, ZIP5, ZIP12 and ZIP13 predicted both shorter OS and PFS. In addition, further investigation from another dataset in TCGA consistently supported that high ZIP12 and ZIP13 expressions were closely related to poor OS in ovarian cancer patients. This is the first attempt to evaluate the prognostic value of ZIP family transporters in ovarian cancer and our results suggested that members of ZIP family may be promising prognostic biomarkers of ovarian cancer.

In light of the finding that ZIP13 expression showed the most significant association with the survival of ovarian cancer patients, herein we mainly focus on exploring the importance of ZIP13 in ovarian cancer metastasis. We found that high level of ZIP13 expression was consistently associated with poor OS of ovarian cancer patients in TCGA dataset. IHC analysis of ovarian cancer TMA slide also revealed that high ZIP13 protein expression was significantly associated with serous carcinoma, lymphatic metastasis, distant metastasis, and poor prognosis. Additionally, ZIP13 served as an independent indicator for patients with ovarian cancer in multivariate analysis. These findings indicated that ZIP13 may represent a novel prognostic marker in ovarian cancer.

It needs to be pointed out that other ZIP transporters, such as ZIP3, ZIP5, ZIP10, ZIP12, ZIP13 and ZIP14 also showed correlation with the survival of ovarian cancer patients in some respects. To our knowledge, the physiological roles of these ZIPs have also been explored in certain types of cancer. ZIP3 was reported to be down regulated in both prostate cancer and pancreatic cancer [22–24]. ZIP5 was overexpressed during esophageal tumorigenesis, and played a vital role in esophageal cancer progression [25]. ZIP10 was involved in the development of breast cancer by forming a heteromer with ZIP6 [26, 27]. ZIP14 was identified as a critical mediator of cachexia development in several metastatic cancers, such as metastatic pancreatic, colon, and breast cancers [28, 29]. However, the importance of these potential markers in ovarian cancer is still unclear. Herein, we provided an overview of the first insights about the relationships between gene expression of these ZIPs and prognostic values in ovarian cancer patients. Although ZIP12 was a major regulator of hypoxia-induced pulmonary vascular remodelling, our study suggested that ZIP12 might also play important roles in ovarian cancer. Interestingly, high ZIP7 expression predicted improved OS of ovarian cancer (Table 1), which was inconsistent with other ZIP transporters. This implied that the precise functions of ZIP7 in ovarian cancer still needs to be addressed. In summary, future studies aimed to investigate the functions of these transporters will provide novel insight into the biology and molecular pathogenesis of ovarian cancer.

ZIP13, as a member of SLC39A/ZIP family, is mainly located in intracellular compartments, especially the Golgi apparatus in a variety of cell types (such as osteoblasts, chondrocytes, pulpal cells and fibroblasts) [13]. However, there is little knowledge regarding the potential roles of ZIP13 in cancer. We for the first time provided evidence that ZIP13 played a crucial role in tumor development and progression. Knockout of ZIP13 in ovarian cancer cells suppressed cell proliferation. The migration and invasion of ovarian cancer cells were also significantly reduced after ZIP13 knockout, as assessed by transwell and wound healing assays. The data from adhesion assays also supported the importance of ZIP13 in promoting cell adhesion. Consistent with the in vitro findings, the in vivo studies also showed that ZIP13 knockout decreased peritoneal metastasis compared with the controls.

Tumor metastasis is a multi-step and complicated biological process, and involves coordinated regulation of cell motility, adhesion, invasion, and survival by numerous signaling pathways [30–32]. Our RNA-seq analysis showed that the downstream targets of ZIP13 included genes involved in ECM organization, cytokine-cytokine receptor interaction, cytokine activity and signaling receptor activity. Most of these genes have been identified to have metastasis-promoting functions, including the FN1, COL4A3, JUP, TNF, CXCL1, and IL6 [33–36]. These findings suggested that ZIP13 may promote ovarian cancer progression via the regulation of ECM and cytokine-mediated signaling pathway.

FAK is a unique non-receptor tyrosine kinase that physically and functionally interacts with Src to regulate cell growth, as well as cell migration and invasion [37, 38]. In fact, FAK is overexpressed in most invasive ovarian cancers and plays a crucial role in ovarian cancer migration and invasion [39]. Plenty of evidence suggests that the Src/FAK signaling pathway can be activated via the interactions with signals from integrins, growth factors, chemokines, or GTPase-activating proteins. Subsequently, multiple downstream signaling cascades are triggered, and ultimately contributes to the development and progression of cancer [40]. For example, The Ras/ERK/ MAPK pathway can be activated by Src/FAK and leads to changes in expression of genes responsible for cell proliferation, survival, and invasion [41, 42]. Transcriptional factor STAT3 can also been induced and shown to directly or indirectly upregulate the expression of genes that are required for cancer progression [43, 44]. In addition, Src and FAK can promote the EMT via disruption of E-cad-dependent cell-cell junctions, and thus enhance tumor cell motility [45, 46].Here, we observed a remarkable decrease of Src and FAK phosphorylation in ZIP13-depleted ovarian cancer cells, and this indicated that ZIP13 may promote ovarian cancer metastasis via Src/FAK signaling pathway. Moreover, the ERK signaling pathway, as well as the expression of some transcriptional factors, such as STAT3, Myc, and Jun, were suppressed when ZIP13 was depleted. The expression of several EMT-associated proteins, including Slug, Snail, N-cad, were also downregulated in ZIP13-deficient cells. In addition, our result suggested a negative correlation between the expressions of ZIP13 and E-cad in both ovarian cells and clinical samples.

Considering the fact that ZIP13 is mainly located in intracellular compartments, ZIP13 is predicted to affect intracellular zinc distribution. We also detected an apparent increase of vesicular zinc in cells with ZIP13 knockout. This result supported a hypothesis that the changes of intracellular zinc distribution induced by ZIP13 underlied the metastasis of ovarian cancer. Interestingly, a series of work conducted by Zhou lab demonstrated that Drosophila ZIP13 (dZIP13, CG7816/ZIP99C) acted as an ER/Golgi-resident iron transporter and functioned in the delivery of iron to the secretory compartments in Drosophila. In addition, dZIP13 level was strongly regulated by iron in a post-translational manner [47, 48]. Our previous work also implied that ZIP13 may play a role in ischemia/reperfusion (I/R) injury through the calcium signaling pathway [49]. In other words, we cannot entirely exclude the possibility that intracellular iron and calcium homeostasis may also be affected by ZIP13 loss in ovarian cancer. Further investigation of these areas is still ongoing to bring new insights into ovarian cancer metastasis.

## Conclusions

In summary, we herein identified that ZIP13, a SLC39/ZIP family member, acts as a major mediator of ovarian cancer metastasis. ZIP13 could regulate intracellular zinc distribution and affect the expression of genes involved in ECM organization and cytokine-mediated signaling pathway. The Src/FAK pathway is subsequently activated and leads to the metastasis of ovarian cancer. Thus, ZIP13 may be a valuable therapeutic target for preventing and treating ovarian cancer metastasis.

## Supplementary Information

**Additional file 1: Figure S1.** Kaplan-Meier survival analysis of ZIP3, ZIP5, ZIP10, ZIP12 and ZIP14 expressions in TCGA cohort. **Figure S2.** ZIP13 protein is overexpressed in ovarian cancer . **Figure S3.** The identification of ZIP13 knockout cells. **Figure S4.** ZIP13 knockout suppresses peritoneal spreading and metastasis of ovarian cancer cells in vivo. **Figure S5.** ZIP13 regulates intracellular zinc distribution. **Figure S6.** Quantitative RT-PCR analysis of genes in the Src/FAK signaling pathway. **Figure S7.** Zinc activates the Src/FAK signaling pathway and regulates metastasis-related genes in ovarian cancer cells. **Figure S8.** Correlation analysis of ZIP13 and target genes in ovarian cancer tissues from TCGA.

**Additional file 2: Table S1.** Clinical properties of the ovarian cancer patients used in the analysis. **Table S2.** Summary of the sequences of ZIP13 sgRNAs and primers for CRISPR. **Table S3.** Primers used in quantitative real-time RT-PCR. **Table S4.** The association of ZIP transporters with overall survival (OS) and progression-free survival (PFS) in patients with serous ovarian cancer. **Table S5.** Correlation of ZIP13 gene expression with survival in different clinical stages in ovarian cancer patients. **Table S6.** Correlation of ZIP13 gene expression with survival in different pathological grades in ovarian cancer patients. **Table S7.** KEGG pathway enrichment analysis of the ZIP13-regulated genes. **Table S8.** GO analysis of the biological processes associated with ZIP13-regulated genes. **Table S9.** GO analysis of the molecular functions associated with ZIP13-regulated genes. **Table S10.** KEGG pathway enrichment analysis of the ZIP13-regulated genes in ovarian cancer patients from TCGA.

## Data Availability

The datasets supporting the conclusions of this article are available from the corresponding author on reasonable request.

## Abbreviations

EMT: Epithelial-mesenchymal transition
CSC: Cancer stem cell
VCP: Valosin-containing protein
HGSOC: High-grade serous ovarian cancer
FBS: Fetal bovine serum
TMA: Tissue microarray
IHC: Immunohistochemistry
CCK-8: Cell counting kit 8
RT-PCR: Quantitative real-time PCR
cDNAs: Complementary DNAs
ECL: Enhanced chemiluminescence
SPF: Specific pathogen-free
RNA-seq: RNA sequencing
BGI: Beijing Genomics Institute
DEGs: Differentially expressed genes
CI: Confidence intervals
HR: Hazard ratio
OS: Overall survival
PFS: Progression-free survival
PPS: Post-progression survival
TCGA: The Cancer Genome Atlas
GEO: Gene Expression Omnibus
FIGO: The international Federation of Gynecology and Obstetrics
ICP-MS: Inductively coupled plasma mass spectrometry
ECM: Extracellular matrixes
I/R: Ischemia/reperfusion
SCD-EDS: Spondylocheirodysplastic-Ehlers-Danlos syndrome
