# Supplementary material for: Zinc transporter SLC39A13/ZIP13 facilitates the metastasis of human ovarian cancer cells via activating Src/FAK signaling pathway
Source: J Exp Clin Cancer Res. 2021 Jun 21;40:199. doi: 10.1186/s13046-021-01999-3 (PMC8215834; doi:10.1186/s13046-021-01999-3)
Supplement: Supplementary file 1 — Additional file 1: Figure S1. Kaplan-Meier survival analysis of ZIP3, ZIP5, ZIP10, ZIP12 and ZIP14 expressions in TCGA cohort. Figure S2. ZIP13 protein is overexpressed in ovarian cancer . Figure S3. The identification of ZIP13 knockout cells. Figure S4. ZIP13 knockout suppresses peritoneal spreading and metastasis of ovarian cancer cells in vivo. Figure S5. ZIP13 regulates intracellular zinc distribution. Figure S6. Quantitative RT-PCR analysis of genes in the Src/FAK signaling pathway. Figure S7. Zinc activates the Src/FAK signaling pathway and regulates metastasis-related genes in ovarian cancer cells. Figure S8. Correlation analysis of ZIP13 and target genes in ovarian cancer tissues from TCGA. [file 13046_2021_1999_MOESM1_ESM.docx]

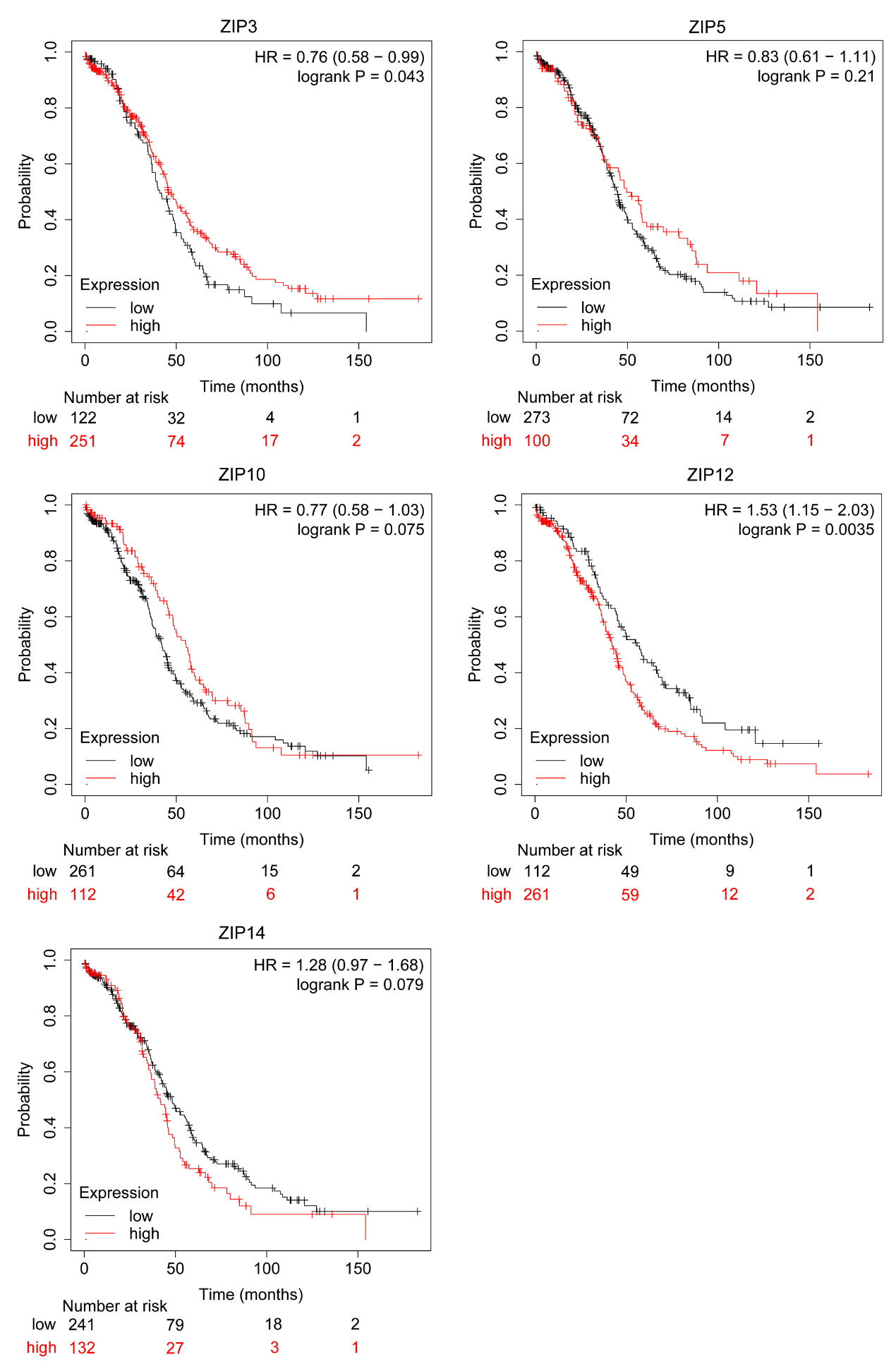


**Fig. S1** Kaplan-Meier survival analysis of ZIP3, ZIP5, ZIP10, ZIP12 and ZIP14 expressions in TCGA cohort.


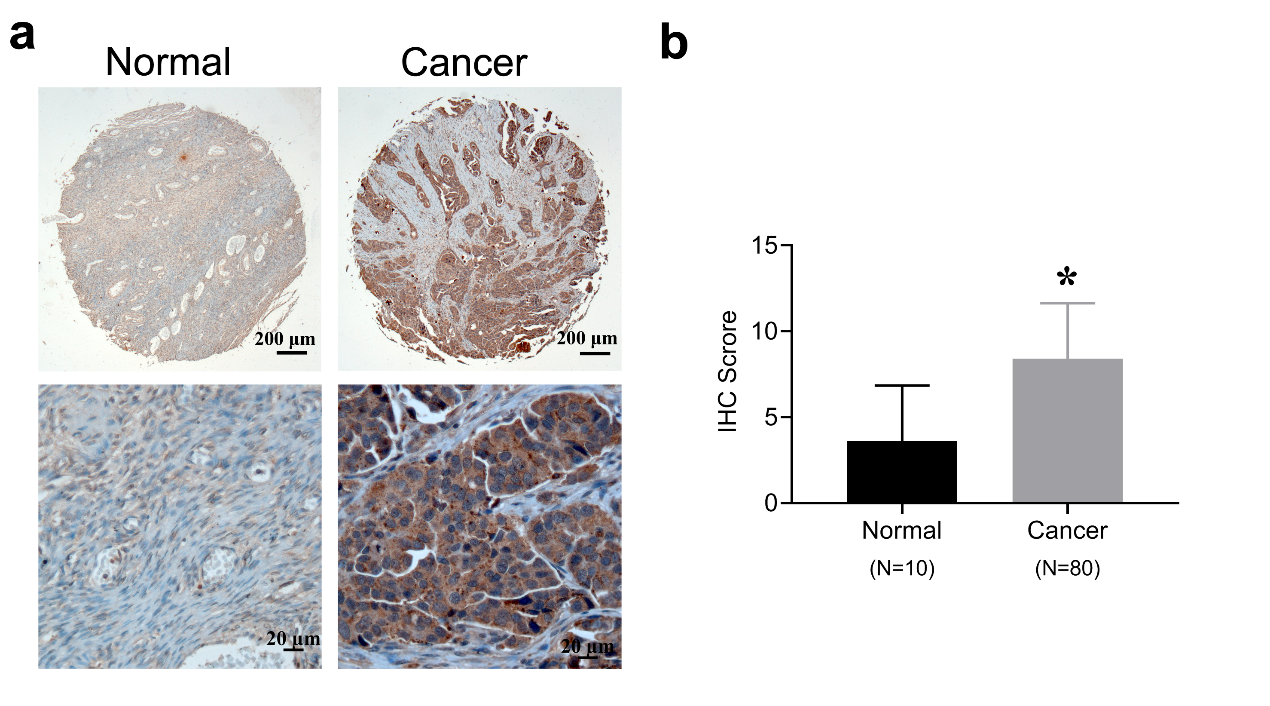


**Fig. S2** ZIP13 protein is overexpressed in ovarian cancer**. a.** Representative immunostaining of ZIP13 in normal tissues and ovarian cancer tissues are shown. **b.** Statistical analysis of ZIP13 IHC scores. * *P* < 0.05


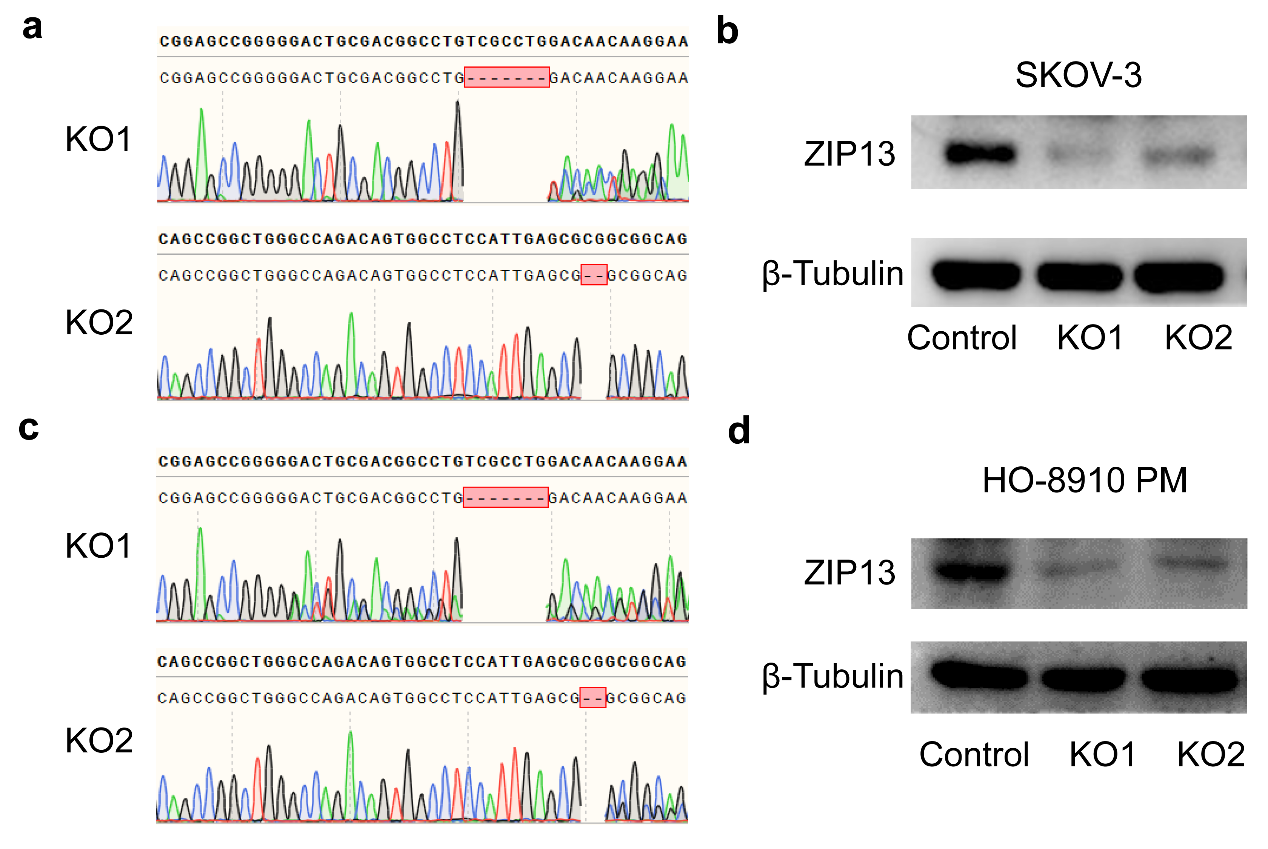


**Fig. S3** The identification of ZIP13 knockout cells. **a, c**. The genomic DNA of cells was amplified and sequenced by the designed primers. **b, d**. The protein expression level of ZIP13 was detected by western blot, and β-tubulin was used as internal control.


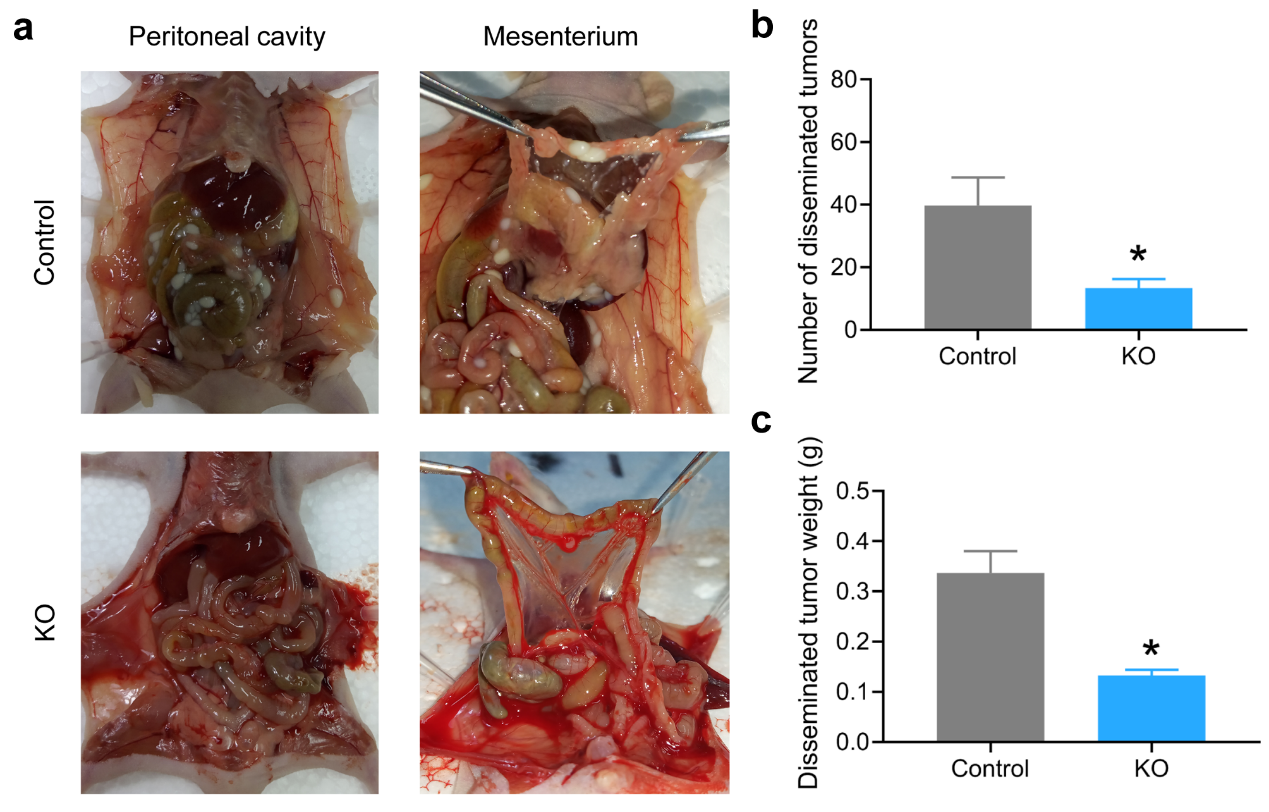


**Fig. S4** ZIP13 knockout suppresses peritoneal spreading and metastasis of ovarian cancer cells in vivo. **a.** HO-8910 PM cell suspensions were injected into peritoneal cavities of BALB/c nude mice (n = 6 mice per group). Representative photos of tumor formation at the peritoneal cavity and mesenterium were shown. **b, c.** The disseminated tumors in the abdominal cavities were estimated and quantified. **P* < 0.05


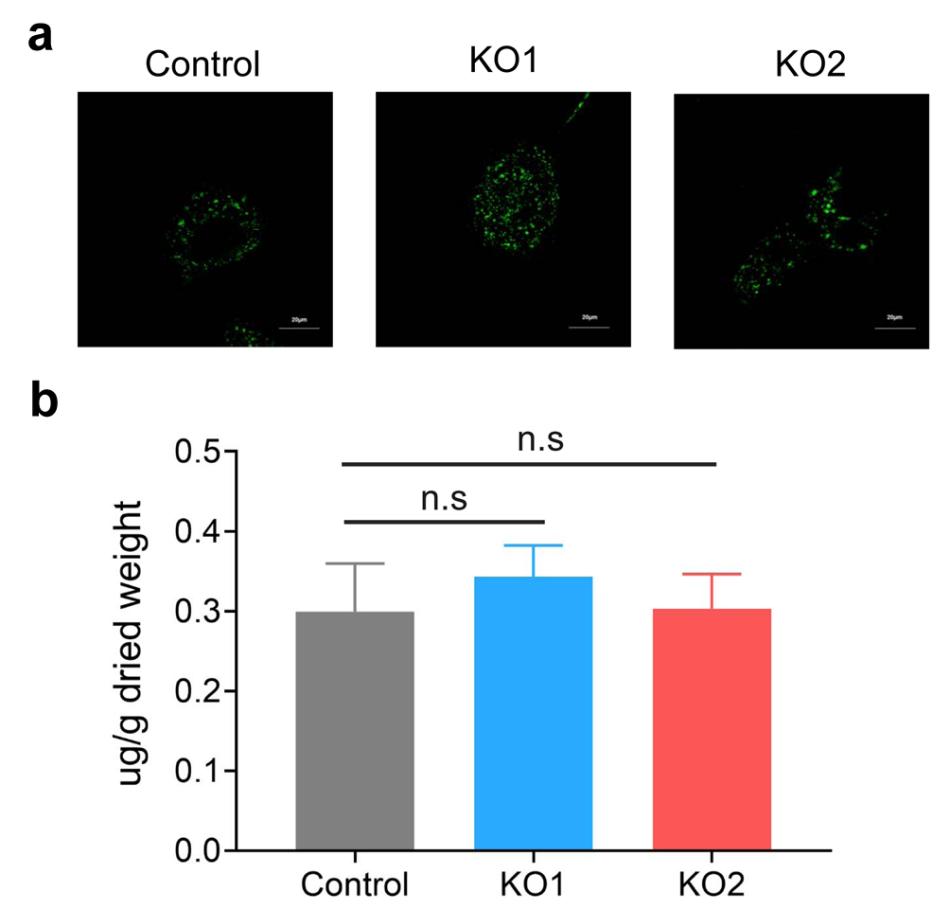


**Fig. S5** ZIP13 regulates intracellular zinc distribution. **a.** SKOV-3 cells were stained with the zinc-specific fluorescent dye, Zinpyr-1 and examined by confocal laser scanning microscope. **b**. Total zinc levels were measured by ICP-MS.

**
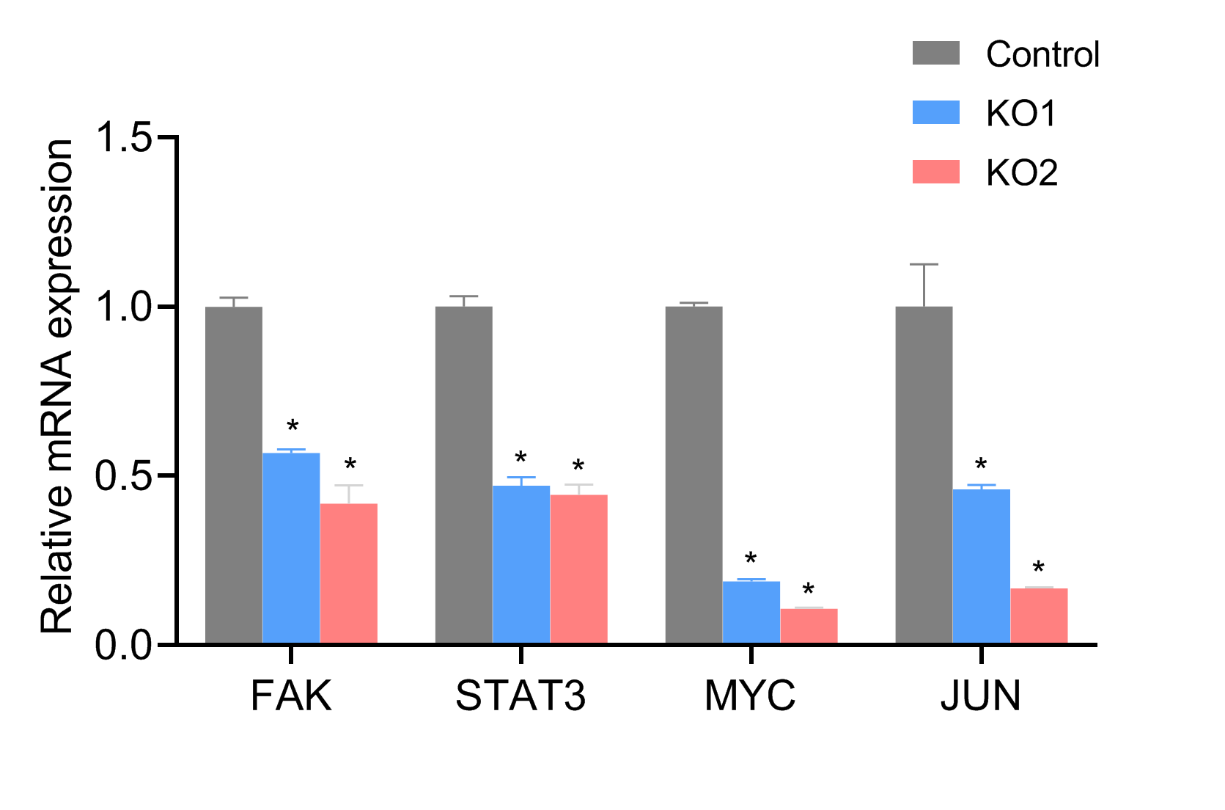
**

**Fig. S6** Quantitative RT-PCR analysis of genes in the Src/FAK signaling pathway. **P* < 0.05


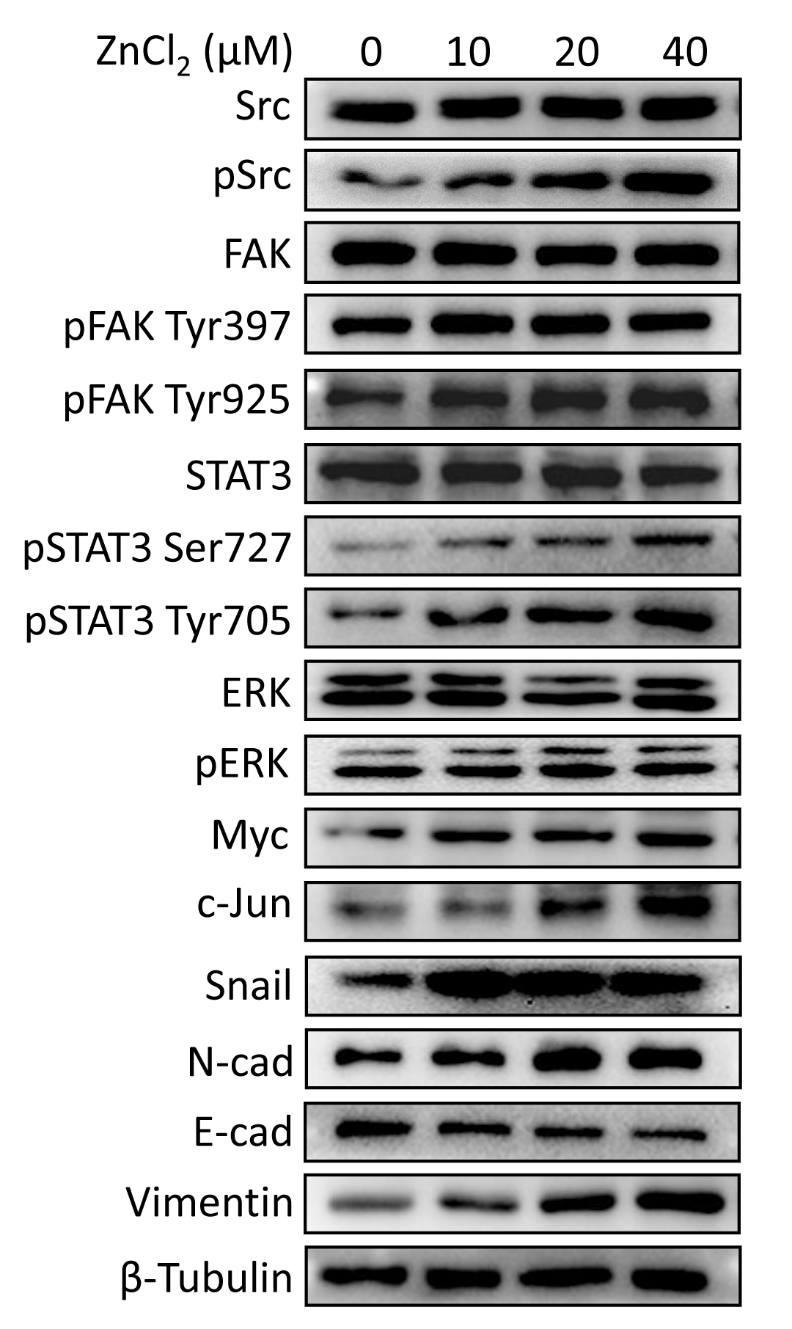


**Fig. S7** Zinc activates the Src/FAK signaling pathway and regulates metastasis-related genes in ovarian cancer cells.


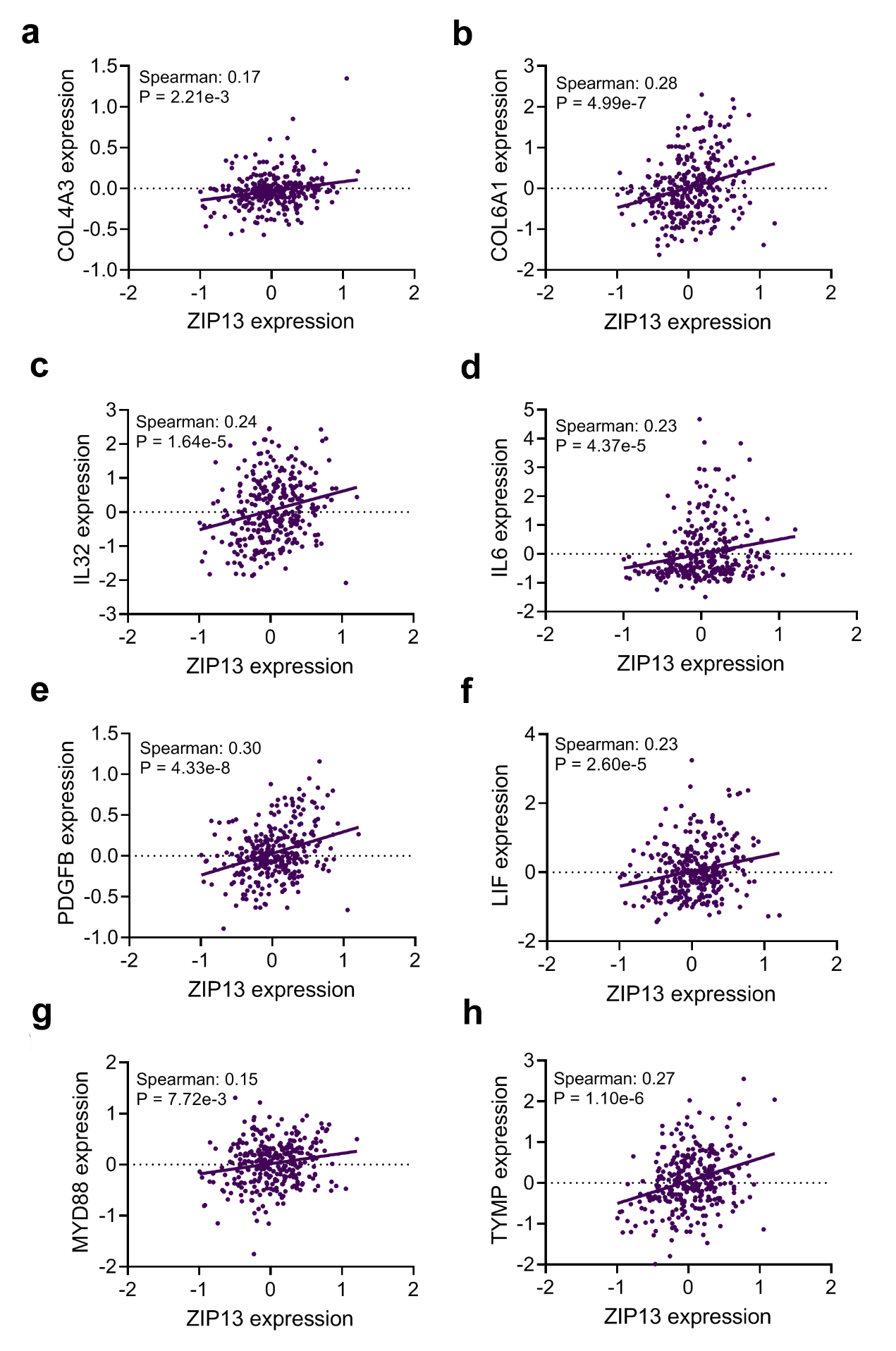


**Fig. S8** Correlation analysis of ZIP13 and target genes in ovarian cancer tissues from TCGA.
